# Two-Dimensional Theoretical Analysis and Experimental Study of Mass Transfer in a Hollow-Fiber Dialysis Module Coupled with Ultrafiltration Operations

**DOI:** 10.3390/membranes13080702

**Published:** 2023-07-27

**Authors:** Chii-Dong Ho, Jr-Wei Tu, Yih-Hang Chen, Thiam Leng Chew

**Affiliations:** 1 Department of Chemical and Materials Engineering, Tamkang University, Tamsui, New Taipei 251301, Taiwanyihhang@mail.tku.edu.tw (Y.-H.C.); 2 Department of Chemical Engineering, Faculty of Engineering, Universiti Teknologi Petronas, Seri Iskandar 32610, Perak, Malaysia; thiamleng.chew@utp.edu.my; 3 CO_2_ Research Center (CO2RES), Institute of Contaminant Management, Universiti Teknologi Petronas, Seri Iskandar 32610, Perak, Malaysia

**Keywords:** hollow-fiber module, dialysis, ultrafiltration, device performance, dialysis-and-ultrafiltration system

## Abstract

This research theoretically and experimentally develops a hollow-fiber dialysis module coupled with ultrafiltration operations by introducing a trans-membrane pressure during the membrane dialysis process, which can be applied to the waste metabolic end products in the human body for improving the dialysis efficiency. The solutes were transported by both diffusion and convection from the concentration driving-force gradient between retentate and dialysate phases across the membrane, compared to the traditional dialysis processes by diffusion only. A two-dimensional modeling of such a dialysis-and-ultrafiltration system in the hollow-fiber dialysis module was formulated and solved using the stream function coupled with the perturbation method to obtain the velocity distributions of retentate and dialysate phases, respectively. The purpose of the present work is to investigate the effect of ultrafiltration on the dialysis rate in the hollow-fiber dialyzer with ultrafiltration operations. A highest level of dialysis rate improvement up to about seven times (say 674.65% under $V_{a}=20\;\mathrm{mL}/\min)$ was found in the module with ultrafiltration rate $V_{w}=10\;\mathrm{mL}/\min$ and membrane sieving coefficient θ=1, compared to that in the system without operating ultrafiltration. Considerable dialysis rate improvements on mass transfer were obtained by implementing a hollow-fiber dialysis-and-ultrafiltration system, instead of using the hollow-fiber dialyzer without ultrafiltration operation. The experimental runs were carried out under the same operating conditions for the hollow-fiber dialyzers of the two experimental runs with and without ultrafiltration operations for comparisons. A very reasonable prediction by the proposed mathematical model was observed.

## 1. Introduction

Membrane dialysis is a membrane technology used widely in Donnan dialysis [1], alcohol reduction of beverages [2], selected acids [3], and hemodialysis [4,5]. Removing waste metabolic end products from the human body is also called artificial kidney [6] in the hemodialysis process, which entails the concentration driving-force difference of the solute transported by diffusion from the feed stream (defined as retentate phase) to the receiving stream (defined as dialysate phase). The theoretical analysis in mass transfer characteristics of membrane dialysis is focused only on the concentration variations of retentate phase. Previous literature [7] assumes solvent not passing through the membrane, in which the velocity distributions in the shell side were solved by using the Happel’s free surface model [8] in a hollow-fiber system without ultrafiltration [9]. However, for the hollow fiber system with ultrafiltration, the velocity distribution is more complex and is developed by a two-dimensional mathematical model to simulate the mass transport in a hollow-fiber artificial kidney, assuming the shell side as a porous medium [10]. The influence of ultrafiltration operations on mass transfer in a hollow-fiber hemodialyzer was discussed by Jagannathan and Shettigar [11,12] and applied in the context of a circular conduit dialyzer [13] and parallel-plate dialyzer with ultrafiltration operations [14]. The treatment time tolerated by patients can be reduced by applying a hemodialysis membrane with ultrafiltration operations in hollow-fiber hemodialyzers [15,16], which is the active cure to elongate the lives of people suffering from end-stage renal diseases (ESRD) [17]. Moreover, a reactive oxygen species of the tannic acid (TA) coating was employed as the functionalized hemodialysis membrane to mitigate oxidative stress [18] in hemodialysis therapy.

The hollow-fiber module provides a higher mass-transfer rate without flooding or loading by implementing a bundle of fiber cell inside the shell side [19]. Studies of experiments [20] and engineering approaches [21] were reviewed on shell-side mass-transfer efficiency in hollow-fiber membrane modules. Many theoretical models explore the mass-transfer phenomena of membrane dialysis on the fundamentals or the design aspects by the packing density with randomly [22] and orderly [23] packed fibers, as well as the dialysate phase velocity distribution [24]. A novel theoretical model for mass transfer of hollow fiber bundles in hollow-fiber hemodialyzers is presented with two non-interpenetrating porous flow zones [25]. Computational fluid dynamics (CFD) was used to study the specific advantages of the efficient device performance in the membrane separation process over conventional separation processes [26]. Furthermore, a non-regenerated recirculating dialysate system was designed to improve the device performance of the hollow-fiber dialyzer [27]. The hollow-fiber dialyzer with ultrafiltration operations to separate urea solute was conducted and illustrated to prove the theoretical predictions of the clearance [28]. Theoretical formulations on the assumption based on the shell-side mass transfer of an ordered fiber array was developed in the present work. The velocity distributions in both retentate and dialysate phases were achieved in terms of the stream functions coupled with the perturbation method [29,30]. While the mathematical formulations were solved numerically by the Crank–Nicolson method, the dialysis efficiency was obtained using retentate and dialysate phase flow rates, ultrafiltration flow rate, membrane sieving coefficient and channel thickness ratio as parameters. The theoretical predictions show that the influence of the ultrafiltration operation dominates dialysis efficiency. The absorption efficiency improvement of the hollow-fiber membrane dialyzer is achieved by implementing ultrafiltration operations under various operating and design conditions. The influences of operating and design parameters on dialysis rate and dialysis efficiency are also delineated. The aims in this work are to develop the two-dimensional mathematical statements of a hollow-fiber membrane dialyzer with operating ultrafiltration and to obtain the theoretical predictions compared with experimental data. The general applicability of such a mathematical formulation was developed similar to the parallel-plate dialysis-and-ultrafiltration system in our previous work [14]. The previous work was confined to a parallel-plate channel while the present paper deals with the conjugated problems in a circular tube with an imaginary shell. The mass-transfer mathematical formulation is somehow different and relatively complicated in analyzing the hollow-fiber membrane dialyzer module in this study. Successful augmentations of membrane dialyzers have been employed to the anion exchange membranes for acid recovery from acidic wastewater [31]. The computational fluid dynamics (CFD) is useful in studying the performance of hollow-fiber membrane contactors for acid gas removal which can decrease the economic cost of experimental works [32]. Some functionalized hemodialysis membranes were used to relieve oxidative stress [18], hemodialysis therapy, and heparin-free hemodialysis [33,34]. Moreover, the anion exchange membranes for acid recovery depends on the dialysis efficiency, membrane selectivity, and distribution coefficient [35]. Results indicated that the ultrafiltration rate plays a significant role in the clearance of the solute [11]. Moreover, the availability of such a two-dimensional mathematical formulation developed here for simulating follow-fiber modules is the main contribution in the present work. The present mathematical treatment could be applied to deal with multi-stream or multi-phase mass-transfer modules in membrane separation processes of industrial engineering design, which have not been solved elsewhere.

## 2. Mathematical Formulations

### 2.1. Dialysis-and-Ultrafiltration in a Hollow-Fiber Membrane Dialyzer Module

**Figure 1:**
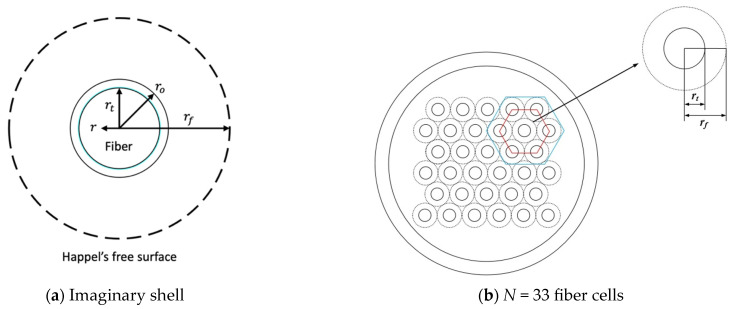
A schematic diagram for the Happel’s free surface model.

A fiber-cell model with the imaginary outer surface of each cell (say the Happel’s free surface) was developed [8,9] to describe the mass transfer behavior between the outer shell feed stream with each fiber cell in the hollow-fiber dialyzer module. Each polysulfone fiber-cell membrane (total $N_{f}=33$ fiber cells inside the hollow-fiber shell tube) with thickness δ (inner radius $r_{t}\;$and outer radius $r_{o}$) is inserted into a shell tube (radius $r_{s}$) of length L to build up the hollow-fiber membrane module, which divides into two open conduits, a fiber-cell inner channel (retentate phase) and annular channel (dialysate phase), as shown in Figure 1, simplified into an imaginary shell/tube unit for each fiber cell. The mathematical modeling was developed by assuming the bundle’s porosity Φ as equal to the uniform packing density, neglecting wall friction on the imaginary free surface and ignoring the velocity distribution across the module radius direction. The imaginary free surface radius$\;r_{f}\;$is defined as:

(1) $$r_{f}=\Phi^{-0.5}r_{o}$$

in which $\Phi =\frac{N_{f}r_{o}^{2}}{\;r_{s}^{2}}$, and thus, Equation (1) was rewritten as

(2) $$r_{f}=\frac{r_{s}}{\;\sqrt{N_{f}}}\;$$

**Figure 2:**
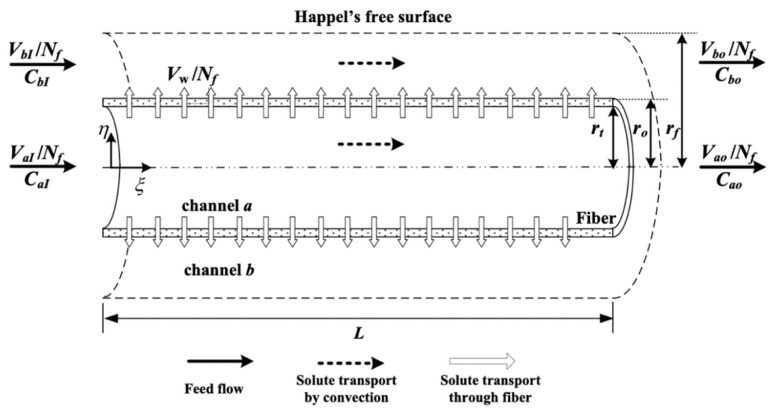
Hollow-fiber dialysis-and-ultrafiltration membrane module.

An ultrafiltration flow for each fiber cell with flow rate $V_{w}/N_{f}$ was operated on the outer boundary of the fiber cell instead of without ultrafiltration operation (say $V_{w}=0$), in which both inlet retentate and dialysate phases flow rates, $V_{aI}/N_{f}$ and $V_{bI}/N_{f}$,were distributed to flow into each fiber cell, leading to dialysis rate improvement. Three mass transfer regimes were schematized to model the hollow-fiber dialysis-and-ultrafiltration membrane module, as shown in Figure 2. The theoretical statement was developed under the following assumptions: (a) Happel’s surface model used to describe the velocity distribution in each fiber cell; (b) isothermal operation and constant physical properties of fluid; (c) steady state and fully developed laminar flow inside the retentate and dialysate phases on entire module; (d) neglecting entrance length and end effects; (e) neglecting longitudinal diffusion; (f) constant membrane sieving coefficient θ.

#### 2.1.1. Velocity Profiles

The velocity profiles were solved with using the continuity equations and momentum balance equations in both fiber cell (channel *a*) and imaginary annulus (channel *b*) as follows:

(3) $$\frac{u_{ar}}{r}+\frac{\partial u_{ar}}{\partial r}+\frac{\partial u_{az}}{\partial z}=0$$

(4) $$\frac{u_{br}}{r}+\frac{\partial u_{br}}{\partial r}+\frac{\partial u_{bz}}{\partial z}=0$$

(5) $$u_{az}\frac{\partial u_{az}}{\partial z}+u_{ar}\frac{\partial u_{az}}{\partial r}=-\frac{1}{\rho }\frac{\partial p_{a}}{\partial z}+\upsilon \left(\frac{\partial^{2}u_{az}}{\partial r^{2}}+\frac{\partial u_{az}}{r\partial r}+\frac{\partial^{2}u_{az}}{\partial z^{2}}\right)$$

(6) $$u_{az}\frac{\partial u_{ar}}{\partial z}+u_{ar}\frac{\partial u_{ar}}{\partial r}=-\frac{1}{\rho }\frac{\partial p_{a}}{\partial r}+\upsilon \left(\frac{\partial^{2}u_{ar}}{\partial r^{2}}+\frac{\partial u_{ar}}{\partial r}-\frac{u_{ar}}{r^{2}}+\frac{\partial^{2}u_{ar}}{\partial z^{2}}\right)$$

(7) $$u_{bz}\frac{\partial u_{bz}}{\partial z}+u_{br}\frac{\partial u_{bz}}{\partial r}=-\frac{1}{\rho }\frac{\partial p_{b}}{\partial z}+\upsilon \left(\frac{\partial^{2}u_{bz}}{\partial r^{2}}+\frac{\partial u_{bz}}{r\partial r}+\frac{\partial^{2}u_{bz}}{\partial z^{2}}\right)\;\;\;$$

(8) $$u_{bz}\frac{\partial u_{br}}{\partial z}+u_{br}\frac{\partial u_{br}}{\partial r}=-\frac{1}{\rho }\frac{\partial p_{b}}{\partial r}+\upsilon \left(\frac{\partial^{2}u_{br}}{\partial r^{2}}+\frac{\partial u_{br}}{\partial r}-\frac{u_{br}}{r^{2}}+\frac{\partial^{2}u_{br}}{\partial z^{2}}\right)$$

The consequential boundary equations of Equations (3)–(8) are

(9) $$\frac{\partial u_{az}\left(0,\;z\right)}{\partial r}=0,\;0\leq z\leq L$$

(10) $$u_{az}\left(r_{t},\;z\right)=0,\;0\leq z\leq L$$

(11) $$u_{az}\left(r,\;0\right)=2\bar{u_{aI}}\left(1-\frac{r^{2}}{r_{t}^{2}}\right),\;0\leq r\leq r_{t}$$

(12) $$-p_{a}+\mu \frac{\partial u_{az}\left(r,\;1\right)}{\partial z}=0,\;0\leq r\leq r_{t}$$

(13) $$u_{ar}\left(r_{t},\;z\right)=u_{br}\left(r_{o},\;z\right),\;0\leq z\leq L$$

(14) $$u_{ar}\left(0,\;z\right)=0,\;0\leq z\leq L$$

(15) $$u_{ar}\left(r,\;0\right)=0,\;0\leq r\leq r_{t}$$

(16) $$\frac{\partial u_{ar}\left(r,\;1\right)}{\partial z}=0,\;0\leq r\leq r_{t}$$

(17) $$u_{bz}\left(r_{o},\;z\right)=0,\;0\leq z\leq L$$

(18) $$u_{bz}\left(r_{f},\;z\right)=0,\;0\leq z\leq L$$

(19) $$u_{bz}\left(r,\;0\right)=2\bar{u_{bI}}\frac{\left(r_{f}^{2}-r_{o}^{2}\right)\left[r_{o}^{2}-r^{2}+2r_{f}^{2}\left(lnr-lnr_{o}\right)\right]}{\left[4r_{o}^{2}r_{f}^{2}-3r_{f}^{4}-r_{o}^{4}+4r_{f}^{4}\left(lnr-lnr_{o}\right)\right]},\;\;r_{o}\leq r\leq r_{f}$$

(20) $$-p_{b}+\mu \frac{\partial u_{bz}\left(r,\;1\right)}{\partial z}=0,\;r_{o}\leq r\leq r_{f}$$

(21) $$\frac{\partial u_{br}\left(r_{f},\;z\right)}{\partial r}=0,\;0\leq z\leq L$$

(22) $$u_{br}\left(r,\;0\right)=0,\;r_{o}\leq r\leq r_{f}$$

(23) $$\frac{\partial u_{br}\left(r,\;1\right)}{\partial z}=0,\;r_{o}\leq r\leq r_{f}$$

in which $\bar{u_{aI}}=V_{aI}/N_{f}\pi r_{t}^{2}$ and $\bar{u_{bI}}=V_{bI}/N_{f}\pi \left(r_{f}^{2}-r_{o}^{2}\right)$ are the average velocities for both inner channel *a* and subchannel *b* (imaginary annulus). The assumptions of Equations (12) and (20) were made with no ultrafiltration flux $V_{w}=0$ at the outlet (*z = L*). Meanwhile, the average ultrafiltration flux transporting through the membrane was evaluated as

(24) $$v_{w}\left(r\right)=\frac{V_{w}}{2\pi rL},\;r_{t}\leq r\leq r_{o}$$

Then, the ultrafiltration flux on both inner and outer surfaces of the membrane were averaged accordingly, say $v_{w0}=V_{w}/2\pi r_{t}L$ and $v_{w1}=V_{w}/2\pi r_{o}L$, respectively. Therefore, Equation (13) was given as follows:

(25) $$u_{ar}\left(r_{t},z\right)=v_{w0}=\mathrm{constant},\;0\leq z\leq L$$

(26) $$u_{br}\left(r_{o},z\right)=v_{w1}=\mathrm{constant},\;0\leq z\leq L$$

The stream function [30] of the fiber-cell channel *a* was generated by the use of the mass conservation in the fiber-cell channel in terms of the dimensionless group $\eta^{*}={\left(r/r_{f}\right)}^{2}$ as

(27) $$\psi_{a}\left(\;\eta^{*},z\right)=\left[r_{t}^{2}\bar{u_{aI}}-2r_{t}v_{w0}z\right]f_{a}\left(\eta^{*}\right)$$

The derivatives of Equation (27) with respect to r and z, respectively, were made to give

(28) $$\frac{\partial \psi_{a}}{\partial r}=ru_{az}\;\mathrm{or}\;\;u_{az}=\left[2\Delta_{f}^{2}\bar{u_{aI}}-4\frac{\Delta_{f}v_{w0}}{r_{s}}z\right]f_{a}^{\prime }\left(\eta^{*}\right)$$

(29) $$\frac{\partial \psi_{a}}{\partial z}=-ru_{ar}\;\mathrm{or}\;\;u_{ar}=\frac{2\Delta_{f}v_{w}}{\sqrt{\eta^{*}}}f_{a}\left(\eta^{*}\right)$$

in which $\Delta_{f}=r_{t}/r_{f}$. Substituting Equations (28) and (29) into Equations (5) and (6) and introducing the perturbation method [30] with the use of appropriate boundary conditions yields the dimensionless forms of velocity distributions of Equations (30) and (31), $u_{a\xi }$ and $u_{a\eta }$, respectively, in axial and radial directions in the inner channel, which were derived in the Appendix A as follows:

(30) $$\;u_{a\xi }=\left[2\Delta_{f}^{2}\bar{u_{aI}}-4\frac{\Delta_{f}v_{w0}L}{r_{f}}\xi \right]\left\{\frac{\left(\Delta_{f}^{2}-\eta^{2}\right)}{\Delta_{f}^{4}}\right.+\lambda_{a}\left[\frac{1}{18\Delta_{f}^{2}}-\frac{\eta^{2}}{4\Delta_{f}^{4}}+\frac{\eta^{4}}{4\Delta_{f}^{6}}-\frac{\eta^{6}}{18\Delta_{f}^{8}}\right]\;\;\;\;\;\;\;\;\;\;\left.+\lambda_{a}^{2}\left[\frac{83}{5400\Delta_{f}^{2}}-\frac{19\eta^{2}}{270\Delta_{f}^{4}}+\frac{11\eta^{4}}{144\Delta_{f}^{6}}-\frac{\eta^{6}}{36\Delta_{f}^{8}}+\frac{\eta^{8}}{144\Delta_{f}^{10}}-\frac{\eta^{10}}{1800\Delta_{f}^{12}}\right]\right\}$$

(31) $$u_{a\eta }=2\Delta_{f}v_{w0}\left\{\frac{1}{\Delta_{f}^{4}}\left(\Delta_{f}^{2}\eta -\frac{\eta^{3}}{2}\right)+\lambda_{a}\left[\frac{\eta }{18\Delta_{f}^{2}}-\frac{\eta^{3}}{8\Delta_{f}^{4}}+\frac{\eta^{5}}{12\Delta_{f}^{6}}-\frac{\eta^{7}}{72\Delta_{f}^{8}}\right]\right.\;\;\;\;\;\;\;\;\;\;+\lambda_{a}^{2}\left[\frac{83\eta }{5400\Delta_{f}^{2}}-\frac{19\eta^{3}}{540\Delta_{f}^{4}}+\frac{11\eta^{5}}{432\Delta_{f}^{6}}-\frac{\eta^{7}}{144\Delta_{f}^{8}}++\frac{\eta^{9}}{720\Delta_{f}^{10}}-\frac{\eta^{11}}{10800\Delta_{f}^{12}}\right]$$

in which $\eta =\frac{r}{r_{f}}$, $\xi =\frac{z}{L}$ and wall Reynolds number of retentate phase $\lambda_{a}=\Delta r_{s}v_{w0}/\upsilon$ for $\left|\lambda_{a}\right|\leq 1$.

Similarly, the dimensionless velocity contributions, $u_{b\xi }$ and $u_{b\eta }$, in the imaginary annulus were obtained by the same solving procedure as characterized by the previous section of inner channel *a*

(32) $$\psi_{b}\left(\eta^{*},z\right)=\left[\left(r_{f}^{2}-r_{o}^{2}\right)\bar{u_{bI}}+2r_{o}v_{w1}z\right]f_{b}\left(\eta^{*}\right)$$

The derivatives of Equation (32) with respect to r and z, respectively, were made to give

(33) $$\frac{\partial \varphi_{b}}{\partial r}=ru_{bz}\;\mathrm{or}\;\;u_{bz}=\left[2{\left(1-\Delta_{f1}\right)}^{2}\bar{u_{bI}}-4\frac{\Delta_{f1}v_{w1}}{r_{f}}z\right]f_{b}^{\prime }\left(\eta^{*}\right)$$

(34) $$\frac{\partial \varphi_{b}}{\partial z}=-ru_{br}\;\mathrm{or}\;\;u_{br}=\frac{2\Delta_{f1}v_{w1}}{\sqrt{\eta^{*}}}f_{b}\left(\eta^{*}\right)$$

in which $f_{b}\left(\eta \right)=f_{b0}\left(\eta \right)+\lambda_{b}f_{b1}\left(\eta \right)$ with $\Delta_{f1}=\frac{r_{o}}{r_{f}},\;\bar{u_{bI}}=\frac{V_{bI}}{\pi \left(r_{f}^{2}-r_{o}^{2}\right)},\;v_{w1}=\frac{V_{w}}{2\pi r_{o}L},\;\;\lambda_{b}=\frac{\Delta_{f1}r_{f}v_{w1}}{\upsilon }$ and the dimensionless forms of velocity profiles of Equations (33) and (34), $u_{b\xi }$ and $u_{b\eta }$, respectively, were obtained as follows:

(35) $$\;u_{b\xi }=\left[2\left(1-\Delta_{f1}^{2}\right)\bar{u_{bI}}+4\frac{\Delta_{f1}v_{w1}L}{r_{s}}\xi \right]\left(f_{b0}^{\prime }\left(\eta \right)+\lambda_{b}f_{b1}^{\prime }\left(\eta \right)\right)$$

(36) $$\;u_{b\eta }=\frac{2\Delta_{f1}v_{w1}}{\eta }\left(f_{b0}\left(\eta \right)+\lambda_{b}f_{b1}\left(\eta \right)\right)$$

where

(37) $$f_{b0}\left(\eta \right)=S_{b0}\left[\frac{\eta^{4}-1}{2}+\left(1-\Delta_{f1}^{2}+ln\Delta_{f1}^{2}\right)\left(\eta^{2}-1\right)-ln\eta^{2}\right]$$

and

(38) $$\begin{array}{c} f_{b1}\left(\eta \right)=\frac{s_{b1}}{2}\eta^{4}-I_{0}\left(\eta^{2}ln\;\eta^{2}-\eta^{2}\right)+I_{1}\eta^{2}+I_{2}\\ \;\;+S_{b0}{}^{2}\left\{\frac{\eta^{2}}{2}\left(-3+2\Delta_{f1}^{2}-2ln\Delta_{f1}^{2}\right)+\frac{\eta^{6}}{72}\left(5-6\Delta_{f1}^{2}+6ln\Delta_{f1}^{2}\right)+\frac{\eta^{8}}{72}\right.\\ \;+\frac{\eta^{4}}{4}\left(27-14\Delta_{f1}^{2}+2\Delta_{f1}^{4}+14ln\Delta_{f1}^{2}-4\Delta_{f1}^{2}ln\Delta_{f1}^{2}+2{\left(ln\Delta_{f1}^{2}\right)}^{2}\right)\\ \;+\frac{\eta^{2}ln\eta^{2}}{12}\left[18-12\Delta_{f1}^{2}+12ln\Delta_{f1}^{2}-\left(36-12\Delta_{f1}^{2}+12ln\Delta_{f1}^{2}\right)\eta^{2}-\eta^{4}\right]\\ \;\left.+\frac{\eta^{2}{\left(ln\eta^{2}\right)}^{2}}{4}\left(-3+2\Delta_{f1}^{2}+2\eta^{2}-2ln\Delta_{f1}^{2}\right)\right\} \end{array}$$

in which $I_{0}$, $I_{1}$, and $I_{2}$ are integration constants, and $S_{b0}$ and $S_{b1}\;$are as follows:

(39) $$\begin{array}{c} I_{0}=\frac{s_{b0}}{\left(-1+\Delta_{f1}^{4}-2ln\Delta_{f1}^{2}\right)}\left[5-\frac{22}{3}\Delta_{f1}^{2}+\frac{31}{12}\Delta_{f1}^{4}-\frac{1}{4}\Delta_{f1}^{8}+\frac{2}{3}ln\Delta_{f1}^{2}+6\Delta_{f1}^{2}ln\Delta_{f1}^{2}\right.\\ \left.\;-\frac{11}{2}\Delta_{f1}^{4}ln\Delta_{f1}^{2}+2\Delta_{f1}^{6}ln\Delta_{f1}^{2}+\frac{3}{2}{\left(ln\Delta_{f1}^{2}\right)}^{2}-\Delta_{f1}^{2}{\left(ln\Delta_{f1}^{2}\right)}^{2}-\Delta_{f1}^{4}{\left(ln\Delta_{f1}^{2}\right)}^{2}-{\left(ln\Delta_{f1}^{2}\right)}^{3}\right] \end{array}$$

(40) $$\begin{array}{c} I_{1}=\frac{S_{b0}}{\left(-1+\Delta_{f1}^{4}-2ln\Delta_{f1}^{2}\right)}\left[\frac{59}{6}\Delta_{f1}^{2}-\frac{209}{24}\Delta_{f1}^{4}-\frac{22}{9}\Delta_{f1}^{6}+\frac{11}{8}\Delta_{f1}^{8}-\frac{1}{18}\Delta_{f1}^{10}+5ln\Delta_{f1}^{2}-\frac{1}{2}\Delta_{f1}^{2}ln\Delta_{f1}^{2}\right.\\ \;\;\;+\frac{47}{6}\Delta_{f1}^{4}ln\Delta_{f1}^{2}-\frac{43}{18}\Delta_{f1}^{6}ln\Delta_{f1}^{2}-\frac{1}{4}\Delta_{f1}^{8}ln\Delta_{f1}^{2}-\frac{1}{12}{\left(ln\Delta_{f1}^{2}\right)}^{2}-\frac{7}{4}\Delta_{f1}^{4}{\left(ln\Delta_{f1}^{2}\right)}^{2}+\frac{3}{2}\Delta_{f1}^{6}{\left(ln\Delta_{f1}^{2}\right)}^{2}\\ \;\;\;\;\;\;\;\;-\frac{1}{2}{\left(ln\Delta_{f1}^{2}\right)}^{3}-\Delta_{f1}^{2}{\left(ln\Delta_{f1}^{2}\right)}^{3}-\frac{1}{2}\Delta_{f1}^{4}{\left(ln\Delta_{f1}^{2}\right)}^{3} \end{array}$$

(41) $$\begin{array}{c} I_{2}=S_{b0}^{2}\left[-\frac{16}{3}+\frac{31}{12}\Delta_{f1}^{2}-\frac{1}{2}\Delta_{f1}^{4}-\frac{31}{12}ln\Delta_{f1}^{2}+\Delta_{f1}^{2}ln\Delta_{f1}^{2}-\frac{1}{2}{\left(ln\Delta_{f1}^{2}\right)}^{2}\right]\\ \;\;\;\;\;\;\;\;\;\;+\frac{S_{b0}}{\left(-1+\Delta_{f1}^{4}-2ln\Delta_{f1}^{2}\right)}\left[-\frac{16}{3}-\frac{47}{12}\Delta_{f1}^{2}+\frac{39}{4}\Delta_{f1}^{4}+\frac{7}{36}\Delta_{f1}^{6}-\frac{3}{4}\Delta_{f1}^{8}+\frac{1}{18}\Delta_{f1}^{10}\right.\\ \;\;\;\;\;\;\;\;\;\;\;\;\;-\frac{53}{4}ln\Delta_{f1}^{2}+3\Delta_{f1}^{2}ln\Delta_{f1}^{2}-\frac{23}{6}\Delta_{f1}^{4}ln\Delta_{f1}^{2}+\frac{7}{18}\Delta_{f1}^{6}ln\Delta_{f1}^{2}+\frac{1}{4}\Delta_{f1}^{8}ln\Delta_{f1}^{2}-\frac{17}{3}{\left(ln\Delta_{f1}^{2}\right)}^{2}\\ \left.+\frac{5}{2}\Delta_{f1}^{2}{\left(ln\Delta_{f1}^{2}\right)}^{2}+\frac{11}{4}\Delta_{f1}^{4}{\left(ln\Delta_{f1}^{2}\right)}^{2}-\frac{3}{2}\Delta_{f1}^{6}{\left(ln\Delta_{f1}^{2}\right)}^{2}-{\left(ln\Delta_{f1}^{2}\right)}^{3}+\Delta_{f1}^{2}{\left(ln\Delta_{f1}^{2}\right)}^{3}+\frac{1}{2}\Delta_{f1}^{4}{\left(ln\Delta_{f1}^{2}\right)}^{3}\right] \end{array}$$

(42) $$S_{b0}=\frac{1}{2{\left(1-\Delta_{f1}^{2}\right)}^{2}+2\;ln\Delta_{f1}^{2}+1-\Delta_{f1}^{4}}$$

(43) $$\begin{array}{c} S_{b1}=\frac{S_{b0}^{2}}{-1+\Delta_{f1}^{4}-2ln\Delta_{f1}^{2}}\left[\frac{2}{3}+\frac{17}{6}\Delta_{f1}^{2}-\frac{29}{4}\Delta_{f1}^{4}+\frac{9}{2}\Delta_{f1}^{6}-\frac{3}{4}\Delta_{f1}^{8}\right]\\ \;+\frac{s_{b0}^{2}ln\Delta_{f1}^{2}}{\Delta_{f1}^{4}-2ln\Delta_{f1}^{2}-1}\left[\frac{91}{6}-17\Delta_{f1}^{2}+3\Delta_{f1}^{4}\right]+\frac{S_{b0}^{2}{\left(ln\Delta_{f1}^{2}\right)}^{2}}{\Delta_{f1}^{4}-2ln\Delta_{f1}^{2}-1}\left[\frac{17}{2}-3\Delta_{f1}^{2}+ln\Delta_{f1}^{2}\right] \end{array}$$

#### 2.1.2. Mass Balance Equations

**Figure 3:**
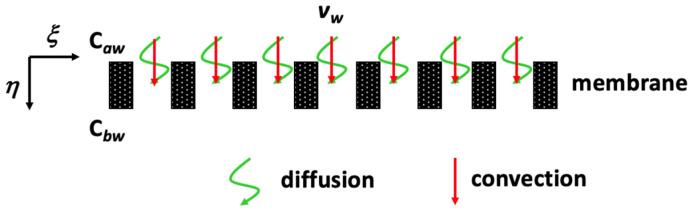
Hollow-fiber membrane dialyzer module with ultrafiltration operations.

The dimensionless mass-transfer equations in the retentate, dialysate, and membrane phases in hollow fiber membrane dialyzer with operating ultrafiltration are derived according to Figure 3 as follows:

(44) $$\frac{u_{a\xi }}{L}\frac{\partial \phi_{a}}{\partial \xi }+\frac{u_{a\eta }}{r_{f}}\frac{\partial \phi_{a}}{\partial \eta }=\frac{D}{r_{f}^{2}}\left(\frac{\partial^{2}\phi_{a}}{\partial \eta^{2}}+\frac{1}{\eta }\frac{\partial \phi_{a}}{\partial \eta }\right),\;0\leq \eta \leq \Delta_{f},\;\;0\leq \xi \leq 1$$

(45) $$\frac{u_{b\xi }}{L}\frac{\partial \phi_{b}}{\partial \xi }+\frac{u_{b\eta }}{r_{f}}\frac{\partial \phi_{b}}{\partial \eta }=\frac{D}{r_{f}^{2}}\left(\frac{\partial^{2}\phi_{b}}{\partial \eta^{2}}+\frac{1}{\eta }\frac{\partial \phi_{b}}{\partial \eta }\right),\;\Delta_{f1}\leq \eta \leq 1,\;0\leq \xi \leq 1$$

(46) $$\frac{v_{w}\left(\eta \right)}{r_{f}}\frac{\partial \phi_{m}}{\partial \eta }=\frac{D_{m}}{r_{f}^{2}}\left(\frac{\partial^{2}\phi_{m}}{\partial \eta^{2}}+\frac{1}{\eta }\frac{\partial \phi_{m}}{\partial \eta }\right),\;\Delta_{f}\leq \eta \leq \Delta_{f1}$$

In which $\phi_{i}=\frac{C_{i}}{C_{aI}}$, and the subscripts *a*, *b*, and *m* refer to the retentate, dialysate, and membrane phases, respectively, $v_{w}\left(\eta \right)=\frac{1}{\eta }\frac{V_{w}}{2\pi r_{f}L}$, $\Delta_{f}\leq \eta \leq \Delta_{f1}$, δ is the membrane thickness, D is the solute diffusivity in the retentate and dialysate phases, $D_{m}$ is solute diffusivity in membrane, ε is the porosity of membrane. The corresponding boundary conditions are

(47) $$\phi_{a}\left(\eta ,\;0\right)=1,\;0\leq \eta \leq \Delta_{f}$$

(48) $$\frac{\partial \phi_{a}\left(0,\;\xi \right)}{\partial \eta }=0,\;0\leq \xi \leq 1$$

(49) $$\theta \phi_{a}\left(\Delta_{f},\;\xi \right)=\phi_{m}\left(\Delta_{f},\;\xi \right),\;0\leq \xi \leq 1$$

(50) $$-D\frac{\partial \phi_{a}\left(\Delta_{f},\;\xi \right)}{\partial \eta }+v_{w0}\phi_{a}\left(\Delta_{f},\;\xi \right)=-D_{m}\frac{\partial \phi_{m}\left(\Delta_{f},\;\xi \right)}{\partial \eta }+v_{w0}\phi_{m}\left(\Delta_{f},\;\xi \right),\;0\leq \xi \leq 1$$

(51) $$\phi_{b}\left(\Delta_{f1},\;\xi \right)=\phi_{m}\left(\Delta_{f1},\;\xi \right),\;0\leq \xi \leq 1$$

(52) $$D\frac{\partial \phi_{b}\left(\Delta_{f1},\;\xi \right)}{\partial \eta }=D_{m}\frac{\partial \phi_{m}\left(\Delta_{f1},\;\xi \right)}{\partial \eta },\;0\leq \xi \leq 1$$

(53) $$\frac{\partial \phi_{b}\left(1,\;\xi \right)}{\partial \eta }=0,\;0\leq \xi \leq 1$$

(54) $$\phi_{b}\left(\eta ,\;0\right)=\phi_{bI},\;\Delta_{f1}\leq \eta \leq 1$$

where θ means that the fraction of solutes is rejected by the membrane.

The solute in membrane phase is simultaneously transported by convection and diffusion due to the dialysis-and-ultrafiltration operation, as shown in Figure 2, and Equation (46) was solved with the use of the boundary conditions of Equations (49) and (51) as

(55) $$\phi_{m}=\frac{\theta \left(\eta^{\alpha_{f}}-\Delta_{f1}^{\alpha_{f}}\right)\phi_{aw}\left(\xi \right)+\left(\Delta_{f}^{\alpha_{f}}-\eta^{\alpha_{f}}\right)\phi_{bw}\left(\xi \right)}{\Delta_{f}^{\alpha_{f}}-\Delta_{f1}^{\alpha_{f}}}$$

in which $\alpha_{f}=V_{w}/2\pi LD_{m}N_{f}$.

The boundary conditions of Equations (50) and (52) were rewritten once Equation (55) was replaced as follows:

(56) $$\frac{\partial \phi_{a}\left(\Delta_{f},\xi \right)}{\partial \eta }=\alpha_{f}\varepsilon \left(\frac{\theta \phi_{aw}\left(\xi \right)-\phi_{bw}\left(\xi \right)}{\Delta_{f}^{\alpha_{f}}-\Delta_{f1}^{\alpha_{f}}}\right)\Delta_{f}^{\alpha_{f}-1}-\left[\frac{r_{f}v_{w0}}{D}\left(\theta -1\right)\right]\phi_{aw}\left(\xi \right),\;0\leq \xi \leq 1$$

(57) $$\frac{\partial \phi_{b\left(\Delta_{f1},\xi \right)}}{\partial \eta }=\alpha_{f}\varepsilon \left(\frac{\theta \phi_{aw}\left(\xi \right)-\phi_{bw}\left(\xi \right)}{\Delta_{f}^{\alpha_{f}}-\Delta_{f1}^{\alpha_{f}}}\right)\Delta_{f1}^{\alpha_{f}-1},\;0\leq \xi \leq 1$$

A model employing two-dimensional mass and momentum balances with the experimental examination was outlined [36], which obtained the complete profile of flow rates along the unit cell of the hollow-fiber module using several approximate one-dimensional models developed in the literature. However, a detailed numerical solution of the two-dimensional problem of possible usefulness was performed in this study. Hence, the two-dimensional concentration distributions in the governing equations, Equations (44) and (45), of a hollow-fiber dialysis-and-ultrafiltration operation were solved with the appropriate boundary conditions. Two limitations of the present research are the assumptions of constant ultrafiltration flux upon the entire module and kept under the uniform packing density during the operation of a hollow-fiber membrane dialyzer module with ultrafiltration. Ultrafiltration flux reduction results from the decrease in effective trans-membrane pressure and all fiber cells may be vibrated and bent due to the convection flow.

### 2.2. Pure Membrane Dialysis in a Hollow-Fiber Membrane Dialyzer Module

A pure hollow-fiber membrane dialysis module without operating ultrafiltration, say $V_{w}=0$, was conducted in transporting the solute by diffusion only in the membrane, as shown in Figure 3. The mass balance equations of the pure membrane dialysis system are the same as the system with dialysis-and-ultrafiltration, except the condition of $V_{w}=0$. The mass balance equations of the pure dialysis system in the retentate, dialysate, and membrane phases are obtained as the following:

(58) $$\frac{u_{a\xi }}{L}\frac{\partial \phi_{a}}{\partial \xi }=\frac{D}{r_{f}^{2}}\left(\frac{\partial^{2}\phi_{a}}{\partial \eta^{2}}+\frac{1}{\eta }\frac{\partial \phi_{a}}{\partial \eta }\right)$$

(59) $$\frac{u_{b\xi }}{L}\frac{\partial \phi_{b}}{\partial \xi }=\frac{D}{r_{f}^{2}}\left(\frac{\partial^{2}\phi_{b}}{\partial \eta^{2}}+\frac{1}{\eta }\frac{\partial \phi_{b}}{\partial \eta }\right)$$

(60) $$\frac{\partial^{2}\phi_{m}}{\partial \eta^{2}}+\frac{1}{\eta }\frac{\partial \phi_{m}}{\partial \eta }=0$$

and the corresponding boundary conditions are

(61) $$\phi_{a}\left(\eta ,\;0\right)=1,\;0\leq \eta \leq \Delta_{f}$$

(62) $$\frac{\partial \phi_{a}\left(0,\;\xi \right)}{\partial \eta }=0,\;0\leq \xi \leq 1$$

(63) $$\theta \phi_{a}\left(\Delta_{f},\;\xi \right)=\phi_{m}\left(\Delta_{f},\;\xi \right),\;0\leq \xi \leq 1$$

(64) $$D\frac{\partial \phi_{a}\left(\Delta_{f},\;\xi \right)}{\partial \eta }=D_{m}\frac{\partial \phi_{m}\left(\Delta_{f},\;\xi \right)}{\partial \eta },\;0\leq \xi \leq 1$$

(65) $$\phi_{b}\left(\Delta_{f1},\;\xi \right)=\phi_{m}\left(\Delta_{f1},\;\xi \right),\;0\leq \xi \leq 1$$

(66) $$D\frac{\partial \phi_{b}\left(\Delta_{f1},\;\xi \right)}{\partial \eta }=D_{m}\frac{\partial \phi_{m}\left(\Delta_{f1},\;\xi \right)}{\partial \eta },\;0\leq \xi \leq 1$$

(67) $$\frac{\partial \phi_{b}\left(1,\;\xi \right)}{\partial \eta }=0,\;0\leq \xi \leq 1$$

(68) $$\phi_{b}\left(\eta ,\;0\right)=\phi_{bI},\;\Delta_{f1}\leq \eta \leq 1$$

Similarly, the two-dimensional concentration distributions of a hollow-fiber dialysis module without ultrafiltration operation in the membrane were obtained following the same procedure performed in the previous section as

(69) $$\phi_{m}=\frac{\theta \left(ln\eta -ln\Delta_{f1}\right)\phi_{aw}\left(\xi \right)+\left(ln\Delta_{f}-ln\eta \right)\phi_{bw}\left(\xi \right)}{ln\Delta_{f}-ln\Delta_{f1}}$$

Substitutions of Equation (69) into the boundary conditions of Equations (50) and (52) yield the rewritten boundary Equations (64) and (66) as

(70) $$\frac{\partial \phi_{a}\left(\Delta_{f},\xi \right)}{\partial \eta }=\frac{\varepsilon }{\Delta_{f}}\left(\frac{\theta \phi_{aw}\left(\xi \right)-\phi_{bw}\left(\xi \right)}{ln\Delta_{f}-ln\Delta_{f1}}\right),\;0\leq \xi \leq 1$$

(71) $$\frac{\partial \phi_{b}\left(\Delta_{f1},\xi \right)}{\partial \eta }=\frac{\varepsilon }{\Delta_{f1}}\left(\frac{\theta \phi_{aw}\left(\xi \right)-\phi_{bw}\left(\xi \right)}{ln\Delta_{f}-ln\Delta_{f1}}\right),\;0\leq \xi \leq 1$$

in which $\phi_{aw}\left(\xi \right)=\phi_{a}\left(\Delta_{f},\xi \right)$ and $\phi_{bw}\left(\xi \right)=\phi_{a}\left(\Delta_{f1},\xi \right)$.

## 3. Dialysis Rate, Dialysis Efficiency, and Dialysis Rate Improvement

The dialysis rates of the hollow-fiber membrane dialysis module with/without ultrafiltration operations are calculated by defining the net removal of the solutes in the retentate phase as

(72) $$M=V_{aI}C_{aI}-V_{ao}C_{ao}$$

where $C_{ao}$ is the average outlet concentration of retentate phase and $V_{ao}$ is the net outlet retentate phase flow rate ($V_{ao}=V_{aI}-V_{w}$). The dialysis efficiency of the hollow-fiber membrane dialysis module with ultrafiltration operation is defined as the ratio of dialysis rate to the maximum solute concentration gradient between both retentate and dialysate phases at the inlet as follows:

(73) $$\chi_{u}=\frac{M}{V_{aI}\left(C_{aI}-C_{bI}\right)}=\frac{1-\left(1-V_{w}/V_{aI}\right)\phi_{a,o}}{1-\phi_{bI}}$$

Furthermore, the dialysis rate improvement $I_{u}\;\left(\%\right)\;$of the hollow-fiber membrane dialysis module with ultrafiltration operation is defined as the percentage increase of the dialysis efficiency $\chi_{u}$ based on the dialysis efficiency of the hollow-fiber membrane dialysis module with no operating ultrafiltration ($V_{w}=0,\;$pure dialysis module)

(74) $$I_{u}\left(\%\right)=\frac{\chi_{u}-\chi_{0}}{\chi_{0}}\times 100\%$$

where the $\chi_{0}$ is the dialysis efficiency of the pure dialysis system without operating ultrafiltration.

## 4. Experimental Runs

**Figure 4:**
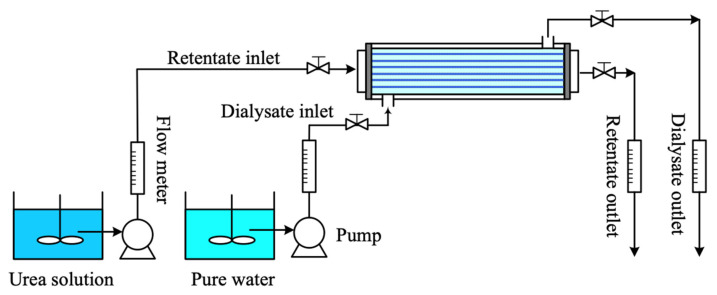
The experimental apparatus of the hollow-fiber dialysis system with ultrafiltration operations.

Figure 4 illustrates the graphical representations of the hollow-fiber membrane dialysis system in the present work. The experiments were carried out to confirm the theoretical predictions obtained from the mathematical models performed in the previous section. A shell tube (length L=30 cm and inner radius $r_{s}=0.45\;\mathrm{cm}$) with 33 membrane fiber cells ($N_{f}=33,\;\mathrm{packing}\;\mathrm{density}\;\Phi =0.2)\;$of membrane thickness (ε=0.01 cm), the inner and outer radius $r_{i}=0.025\;\mathrm{cm}$ and $r_{o}=0.065\;\mathrm{cm}$, respectively, was used in the experiments, in which the membrane was made of MWCO = 10,000 (Xampler UFP-10-C-3MA, Cytiva, Chicago, IL, USA). The solute of urea (MW = 60.06, Kokusam Chemical Works, Yokohama-City, Kanagawa, Japan) was dissolved in pure water of inlet 2 M concentration flowing into the retentate phase while pure water flowed through the dialysate phase with solute diffusivity $D=8\times 10^{-6}\;{\mathrm{cm}}^{2}/s$. The density and the viscosity of pure water at 25 °C were $\rho =0.997\;g/{\mathrm{cm}}^{3}$ and $\mu =8.94\times 10^{-3}\;g/\mathrm{cm}\cdot s$, respectively, while the density and the viscosity of 2M urea solution at 25 °C were $\rho =1.026\;g/{\mathrm{cm}}^{3}$ and $\mu =9.75\times 10^{-3}\;g/\mathrm{cm}\cdot s$, respectively. The retentate phase flow rate ($V_{a}=20,\;25,\;30,\;35,\;40\;\mathrm{mL}/\min$), dialysate phase flow rate ($V_{b}=300\;\mathrm{mL}/\min$), and ultrafiltration rate ($V_{b}=0,\;5,\;10\;\mathrm{mL}/\min$) were the specified operating parameters. The inlet and outlet retentate phase flow rates were regulated to conduct the ultrafiltration rate by the specified operating conditions. The net amount of both flow rates was equal to the setting ultrafiltration rate. The urea concentration in the outlet retentate stream was collected and measured using a UV detector (Unicam UV 300, UV–Visible Spectrometer, LightMachinery, Tampa, FL, USA) for each 5 min interval until the urea concentration was unchanged at steady state. Then, the dialysis efficiency was calculated and recorded. Repeated runs were conducted under the same operating conditions to ensure reproducibility of the experimental results. Comparisons were made between the experimental runs and the theoretical predictions.

## 5. Results and Discussions

The laminar hollow-fiber dialyzer with ultrafiltration operations was investigated theoretically and experimentally to enhance the dialysis efficiency and dialysis rate improvement. The agreement between experimental results and theoretical predictions is promising. The dialysis efficiency of various elements including membrane sieving coefficients, ultrafiltration rates, and retentate phase flow rates were thoroughly compared.

### 5.1. The Numerical Solutions of the Crank–Nicolson Method Validated by Convergence Tolerance

Discretization of the mass balances equations of Equations (44), (45), (58) and (59) and boundary conditions uses the Crank–Nicolson method in both retentate and dialysate phases for hollow-fiber membrane dialysis along the flowing direction with and without ultrafiltration operations, respectively. The 1st order and 2nd order derivatives of partial differential equations were discretized and transformed with routines incorporating the finite difference algorithm as follows:

(75) $$\frac{\partial \phi_{a}}{\partial \xi }=\frac{\phi_{p+1}^{n}-\phi_{p}^{n}}{s}\;\mathrm{and}\;\frac{\partial \phi_{b}}{\partial \xi }=\frac{\phi_{p+1}^{j}-\phi_{p}^{j}}{s}$$

(76) $$\frac{\partial \phi_{a}}{\partial \eta }=\frac{1}{2}\left[\frac{\phi_{p+1}^{n+1}-\phi_{p+1}^{n-1}}{2k}+\frac{\phi_{p}^{n+1}-\phi_{p}^{n-1}}{2k}\right]\;\mathrm{and}\;\frac{\partial \phi_{b}}{\partial \eta }=\frac{1}{2}\left[\frac{\phi_{p+1}^{j+1}-\phi_{p+1}^{j-1}}{2k}+\frac{\phi_{p}^{j+1}-\phi_{p}^{j-1}}{2k}\right]$$

(77) $$\frac{\partial^{2}\phi_{a}}{\partial \eta^{2}}=\frac{1}{2}\left[\frac{\phi_{p+1}^{n+1}-2\phi_{p+1}^{n}+\phi_{p+1}^{n-1}}{k^{2}}+\frac{\phi_{p}^{n+1}-2\phi_{p}^{n}+\phi_{p}^{n-1}}{k^{2}}\right]\;\mathrm{and}\;\frac{\partial^{2}\phi_{b}}{\partial \eta^{2}}=\frac{1}{2}\left[\frac{\phi_{p+1}^{j+1}-2\phi_{p+1}^{j}+\phi_{p+1}^{j-1}}{k^{2}}+\frac{\phi_{p}^{j+1}-2\phi_{p}^{j}+\phi_{p}^{j-1}}{k^{2}}\right]$$

**Figure 5:**
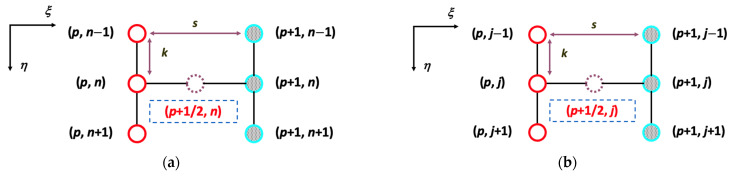
The spaced grids of the Crank–Nicolson method. (**a**) spaced grids in retentate phase; (**b**) spaced grids in dialysate phase.

The scheme of spaced grids (s in ξ-direction and k in η-direction) with node numbers in η-direction are N and J for retentate and dialysate phases, respectively, and the node numbers in ξ-direction are P for both retentate and dialysate phases, as presented in Figure 5. We obtained the matrix form of the resulting equations to solve the formula numerically.

The node numbers in η-direction are N and J grids for retentate and dialysate phases, respectively, and the node numbers in ξ-direction are P grids for both retentate and dialysate phases. The calculated results show that the comparisons with two step sizes in η-direction (N and J) and ξ-direction (P) agree reasonably well in the hollow-fiber dialysis-and-ultrafiltration system as in the illustrations. The Crank–Nicolson method used an average of approximation in J and J+1 row instead of J+1 only, and developed different approximations at the midpoint of the z direction increment to provide a higher order accuracy. The advantages of using the Crank–Nicolson scheme guide the development of improvements to restrict the number of z directions employed in a finite difference approximation and to advantageously to restrict the difference equations leading to a tridiagonal matrix. Obviously, the Crank–Nicolson method is a significant improvement over the forward and backward algorithms. Upon approximating the 2nd derivative of Equations (44) and (45) and Equations (58) and (59) for with and without operating ultrafiltration in the *J* + 1 (or *N* + 1) row instead of the *J* (or *N*) row, one obtains the resultant simultaneous equations in matrix forms to specify the node numbers in η-direction with *N* and *J* for retentate and dialysate phases, respectively. The central difference was used for the boundary conditions. The matrix taken in a tridiagonal form demonstrates that at each step of marching process, it is necessary to explicitly solve a set of simultaneous linear equations for unknowns, thereby eliminating any matrix operations. The numerical solution proceeded successively and converged when the relative error within the allowable convergence tolerance, and hence, dialysis efficiency and dialysis rate improvement were obtained in comparisons between the systems with/without ultrafiltration operations, which the convergence tolerances of the calculated solutions were shown in Table 1.

Moreover, the zero and 1st order perturbation methods were applied to the velocity distributions of $u_{b\xi }$ in the hollow-fiber dialysis-and-ultrafiltration system with the convergence tolerances, as seen from Table 2 for comparisons. The chosen order of perturbation methods indicated in Table 2 are the first two dominant terms in the operating systems due to achieving acceptable convergence tolerance. The results show that only the zero order perturbation in the dialysate phase is required in calculation procedures.

Furthermore, some results of Reynolds number in both retentate and dialysate phases were estimated, respectively, and presented in Table 3 to ensure the system was operating under steady state and fully developed laminar flow. Therefore, the range of Reynolds number in the retentate phase was within 19.23–51.29 under the flow rates 20–40 mL/min while the Reynolds number in the dialysate phase was within 12.82–84.91 for the flow rates under 100–300 mL/min. The operation conditions of both retentate and dialysate phases were confirmed under laminar flow patterns.

### 5.2. Average Concentration Distributions

**Figure 6:**
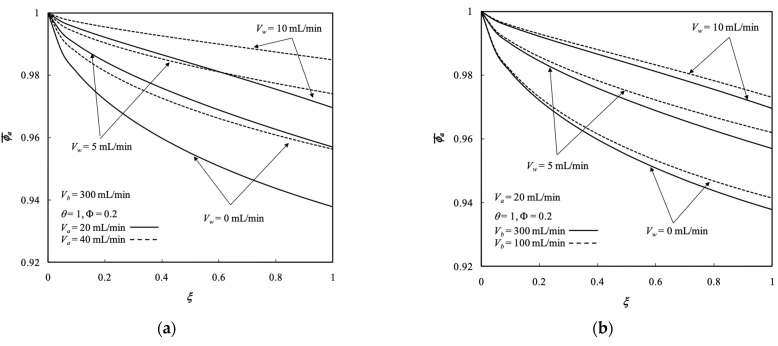
Average concentration distribution of the retentate phase under various ultrafiltration rates. (**a**) with retentate phase flow rate as a parameter; (**b**) with dialysate phase flow rate  as a parameter.

**Figure 7:**
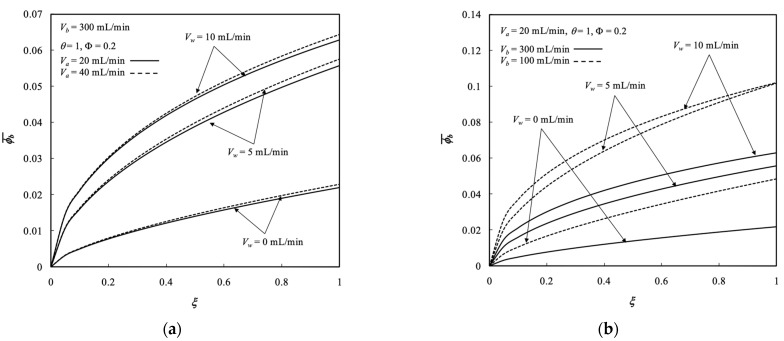
Average concentration distribution of the dialysate phase under various ultrafiltration rates. (**a**) with retentate phase flow rate as a parameter; (**b**) with dialysate phase flow rate  as a parameter.

The effects of ultrafiltration rate and both retentate and dialysate phase flow rates on the average concentration of both retentate and dialysate phases were demonstrated in Figure 6 and Figure 7, respectively. Each legend for those profiles was the transversal average concentration distribution with respect to the longitudinal location. Average concentration distributions in both retentate and dialysate phases increased with the ultrafiltration rate and retentate phase flow rate but decreased with the dialysate phase flow rate, as demonstrated in Figure 6 and Figure 7, respectively, which indicated that the solute was moved from the retentate phase to dialysate phase. The large residence time resulted in a sufficient time to transport solutes from retentate phase to dialysate phase. The curves of $V_{w}=0$ in Figure 6 and Figure 7 denote the pure dialysis system without ultrafiltration operation.

Removing waste metabolic end products (such as the solute of urea) from the human body was conducted in the hemodialysis process for purifying blood. The solute (in the present work of membrane sieving coefficient θ=1 without rejection) associated with solvent (pure water) is transported by mass-transfer diffusion and convection from the feed stream (defined as retentate phase) to the receiving stream (defined as dialysate phase) simultaneously. The larger ultrafiltration rate leads to an increased amount of both solute (urea) and solvent (pure water) in the retentate phase transported through the membrane of the present work, in which the solute and solvent were received by the dialysate phase. Therefore, the dialysis-and-ultrafiltration operation could remove a larger amount of solute from the retentate phase as well as the solvent but may not lessen the average concentration of retentate phase, as observed in Figure 6 and Figure 7.

**Figure 8:**
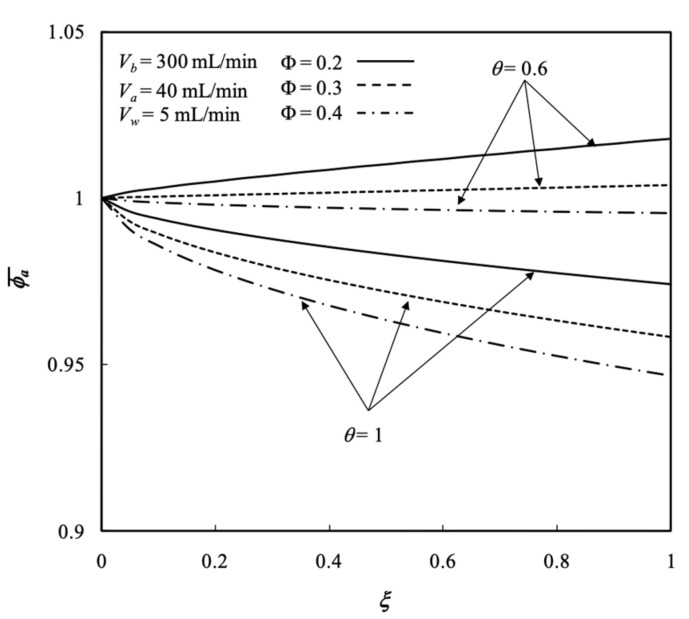
Average concentration distribution of retentate phase with ultrafiltration rate and packing density as parameters.

Furthermore, the influences of the membrane sieving coefficient θ and packing density Φ on the average concentration distribution of the retentate phase were inspected considering the total amount of solute removal, as demonstrated in Figure 8. Figure 8 shows the average concentration distribution of the retentate phase decreases with both the membrane sieving coefficient and packing density. The lower packing density with a smaller number of fiber cells were inserted into the shell, the total amount of mass transfer area ($2\pi r_{t}N_{f})\;$decreased to a lesser extent. Meanwhile, the smaller membrane sieving coefficient deters the mass transferring rate of the solute through the membrane.

### 5.3. Average Outlet Concentrations

**Figure 9:**
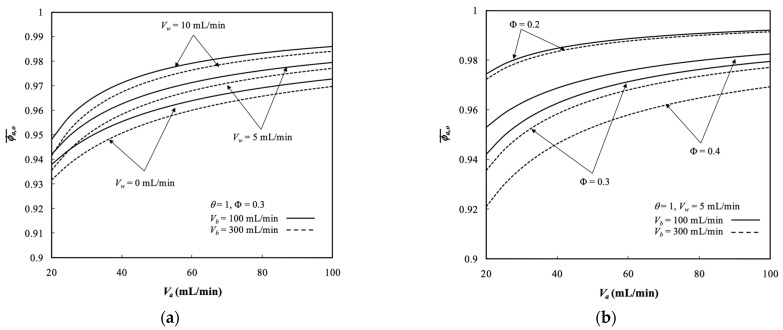
Average outlet concentration distribution of the retentate phase with $V_{w}$, Φ, and $V_{b}$ as parameters. (**a**) with $V_{w}$ and $V_{b}$ as parameters; (**b**) with$\;\Phi \;\mathrm{and}\;V_{b}\;$ as parameters.

The average outlet concentrations of retentate phase $\bar{\phi_{a,o}}$ were indexed to assess the performance of membrane dialysis modules, which were examined with ultrafiltration rate, packing density, and both retentate and dialysate phase flow rates as parameters, as indicated in Figure 9. The results revealed the average outlet concentrations of retentate phase $\bar{\phi_{a,o}}$ decreased with dialysate phase flow rate owing to a larger concentration gradient across the membrane resulting in more solute transporting through the membrane. The average outlet concentration increased with ultrafiltration rate but decreased with packing density due to decreasing residence time of the retentate phase (say $V_{aI}/N_{f}$ with a lesser $N_{f}$).

### 5.4. Dialysis Rate, Dialysis Efficiency, and Dialysis Rate Improvement

**Figure 10:**
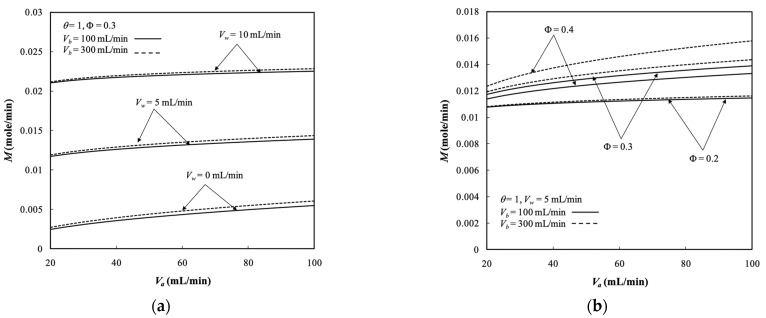
Dialysis rate with $V_{w}$, Φ, and $V_{b}$ as parameters. (**a**) with ultrafiltration rate  as a parameter; (**b**) with packing density as a parameter.

Diffusion through the membrane due to the existing the concentration gradient across both retentate and dialysate phases and convection flow owing to operating the ultrafiltration flux are two mass transfer mechanisms for transporting solutes through the membrane in the dialyzer system. The dialysis rate M of the hollow-fiber module under ultrafiltration operation is determined by Equation (69) and presented in Figure 10. The dialysis rate M in the hollow-fiber dialysis-and-ultrafiltration system increased with the ultrafiltration rate and dialysate phase flow rate. It contributed to increased concentration gradient and thus a higher mass diffusion was achieved. Moreover, the dialysis rate was higher than that in the system with respect to a pure dialysis system (say $V_{w}=0$ without operating ultrafiltration), as shown by Figure 10a. Figure 10b denotes that the dialysis rate M also increased with the packing density, which increased the convective mass-transfer area ($2\pi r_{t}N_{f})$ along with more fiber cells. Figure 10a,b indicates that the dialysis rate *M* increased with both retentate and dialysate phase flow rates, which contributed to the average fluid velocity in both retentate and dialysate phase associated with higher convective mass-transfer coefficients.

**Figure 11:**
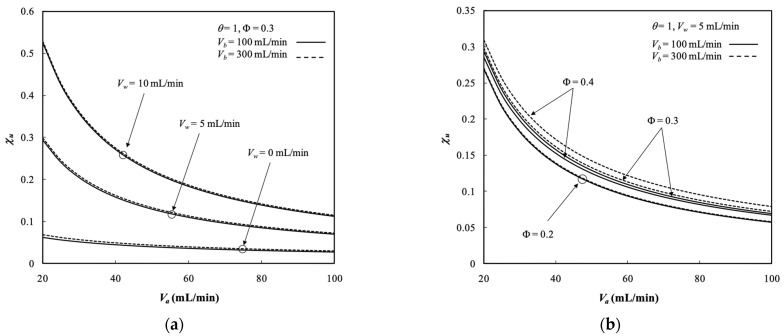
Dialysis efficiency under various ultrafiltration rates and packing densities. (**a**) with ultrafiltration rate as a parameter; (**b**) with packing density as a parameter.

The dialysis efficiency $\chi_{u}$ of the hollow-fiber dialysis-and-ultrafiltration system is expressed in terms of the ratio of the dialysis rate to the maximum solute removal at the entrance, as illustrated in Figure 11a.

The dialysis efficiency $\chi_{u}$ increased significantly with the ultrafiltration rate, especially under the lower dialysate phase flow rate but decreased slightly with the dialysate phase flow rate according to the definition of Equation (73), as shown by Figure 11a. Figure 11b shows that the dialysis efficiency $\chi_{u}$ also increased with the packing density. The more fiber cells implemented with a larger mass transfer area, the greater the dialysis efficiency achieved. Moreover, Figure 11a,b shows that the dialysis efficiency $\chi_{u}$ increased with the dialysate phase flow rate but decreased with the retentate phase volumetric flow rate.

The relative percentage increment of the dialysis rate improvements $I_{u}\left(\%\right)$ in hollow-fiber dialysis-and-ultrafiltration module were evaluated with respect to the module without ultrafiltration operation (a pure dialysis operation). The dialysis rate improvements $I_{u}\left(\%\right)$ of the dialysis rate improvement in the hollow-fiber dialysis-and-ultrafiltration module were estimated and presented in Table 4 for the membrane sieving coefficient θ, ultrafiltration rate $V_{w}$, and both retentate and dialysate flow rates ($V_{a}$ and $V_{b}$). The dialysis rate improvements $I_{u}\;\left(\%\right)$ increased with the $V_{w}$ due to the increase of both mass-transfer diffusion and convective through the membrane from the retentate phase to the dialysate phase. The results also showed that the dialysis rate improvements decreased with both $V_{a}$ and $V_{b}$. However, the highest dialysis rate improvement up to about seven times (say 674.65% under $V_{a}=20\;\mathrm{mL}/\min)\;$was obtained as compared to the counterpart in the system without operating ultrafiltration, as seen from Table 4. The dialysis rate improvements $I_{u}\left(\%\right)$ decreased with the retentate phase flow rate. The solutes were transported through the membrane via diffusion and convection simultaneously in a dialysis-and-ultrafiltration system; however, the effect of convection on the dialysis rate was overwhelmed by the increasing of retentate phase flow rate owing to strengthening the mass-transfer convective coefficient. Therefore, the contribution of the ultrafiltration effect on the dialysis rate wanes. Moreover, the relative percentage increment of $I_{u}\left(\%\right)$ was generated with a larger membrane sieving coefficient θ compared to the pure dialysis without operating ultrafiltration, as indicated in Table 4, especially for a comparatively higher ultrafiltration rate. In other words, the hollow-fiber dialyzer with ultrafiltration operations transported a greater amount of solutes through the membrane associated with a higher dialysis rate. Generally, operating with ultrafiltration rate results in a considerable enhancement in augmenting the dialysis rate in the hollow-fiber module. Therefore, the useful graphical presentations create a better understanding of how a suitable selection of operation conditions can help accomplish a higher device performance in the hollow-fiber dialysis-and-ultrafiltration system.

### 5.5. Experimental Results

Experiments of the hollow-fiber dialyzer with operating ultrafiltration were conducted to validate the theoretical prediction of the two-dimensional mathematical model. The dialysis efficiency of a hemodialyzer using the urea solute in the experiments was defined by the clearance rate of urea [28]. Due to the small molecular weight of urea (MW = 60.06), it can be considered that the urea can freely pass through the composite membrane with MWCO = 10,000 in the present study, i.e., the membrane sieving coefficient of urea was θ=1 in all experiments. The percentage error deviation of the experimental resulting from the theoretical predictions was calculated using the following definition as:

(78) $$Er\left(\%\right)=\frac{\left(\phi_{a,o}^{Theo}-\phi_{a,o}^{Exp}\right)}{\phi_{a,o}^{Exp}}\times 100\%$$

where $\phi_{a,o}^{Theo}$ and $\phi_{a,o}^{Exp}$ are the number of theoretical predictions and experimental results of outlet concentrations in retentate phase, respectively. The comparisons of the theoretical predictions and experimental results for $V_{b}=300\;\mathrm{mL}/\min$ and Φ=0.2 are illustrated in Table 5 and the largest percentage error was of 5.89%.

**Figure 12:**
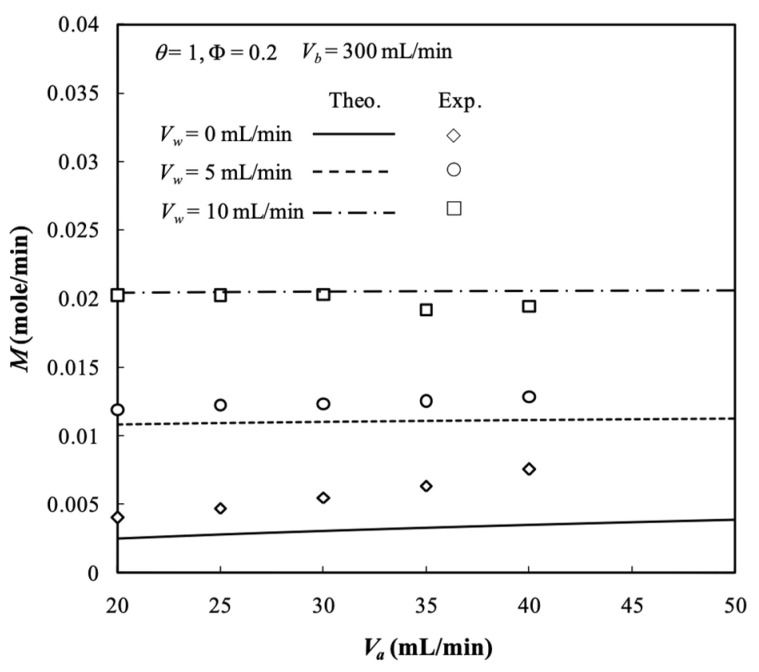
Theoretical and experimental results of dialysis rate with ultrafiltration rate as a parameter.

The experimental results and theoretical predictions of dialysis rate M are shown in Figure 12 and the accuracy deviations of the outlet concentration between experimental data and theoretical results that fall within 0.24% ≤Er≤ 5.89% were calculated in Table 5. Therefore, qualitative and quantitative agreements were reached in the hollow-fiber dialyzer with ultrafiltration operations related with those calculated deviation trends.

### 5.6. Effects of the Membrane Sieving Coefficient on Hollow-Fiber Dialyzer Performance

**Figure 13:**
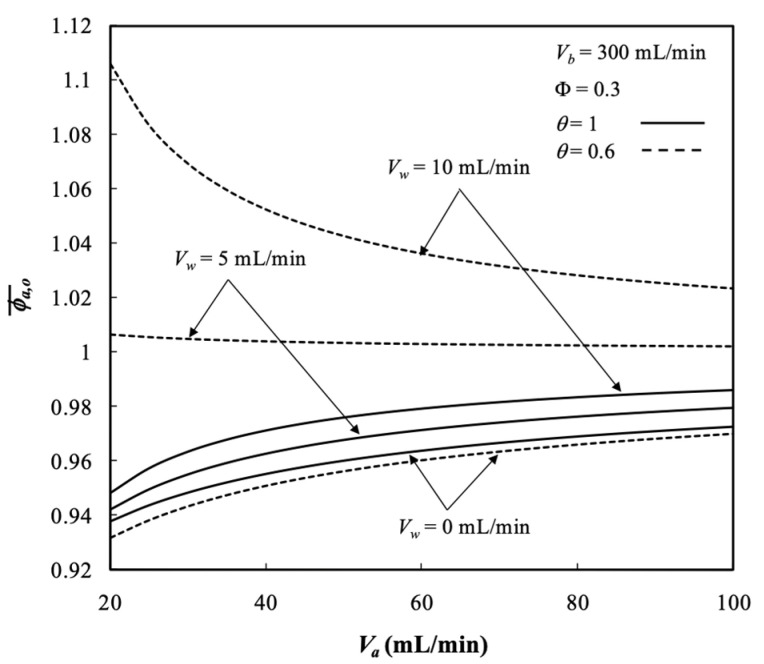
Average outlet concentration distribution of the retentate phase with the membrane sieving coefficient and ultrafiltration rate as parameters.

The solutes are transported liberally through the membrane while the membrane sieving coefficient equals to 1$\;\left(\mathrm{say}\;\theta =1\right)$. These are partially rejected by the membrane while the membrane sieving coefficient is less than 1 (θ<1). Figure 13 shows the effect of the membrane sieving coefficient on the average concentration distribution of the retentate phase. The average outlet concentrations of retentate phase $\bar{\phi_{a,o}}$ decreased with the membrane sieving coefficient but increased with ultrafiltration rate, as seen from Figure 13.

**Figure 14:**
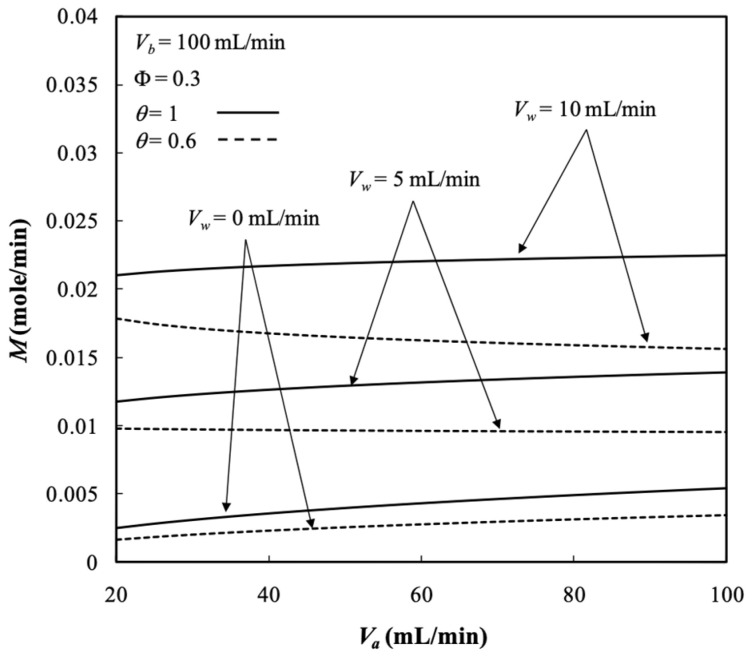
Dialysis rate with the membrane sieving coefficient and dialysate phase flow rate as parameters.

The membrane sieving coefficient plays a significant role in increasing the dialysis rate of mass diffusing through the membrane, and thus, the dialysis rate M increases with the membrane sieving coefficient as well as the ultrafiltration rate and dialysate phase flow rate, as demonstrated in Figure 14. It is obvious that the larger amount of solutes was transported through the membranes under the larger membrane sieving coefficient, ultrafiltration rate, and dialysate phase flow rate.

**Figure 15:**
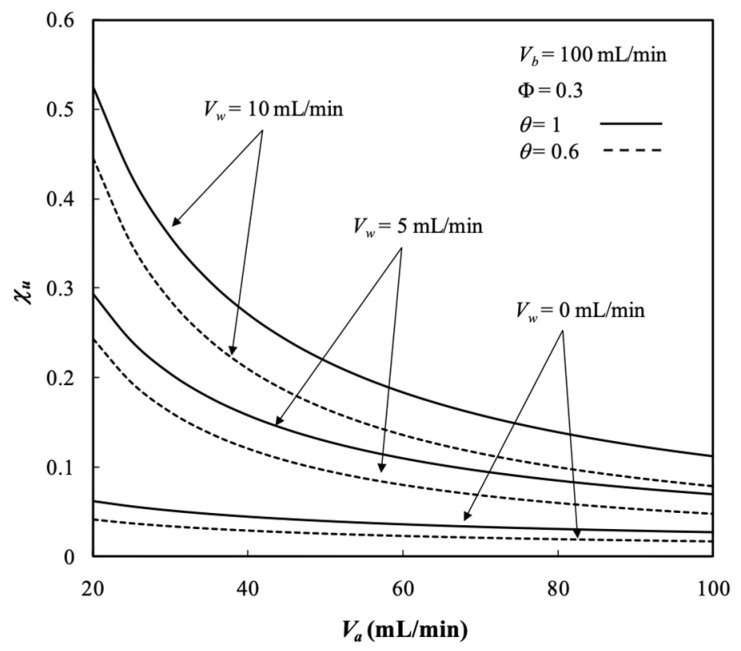
Dialysis efficiency with the membrane sieving coefficient and ultrafiltration rate as parameters.

Furthermore, the influence of the membrane sieving coefficient on the dialysis efficiency $\chi_{u}$ is presented in Figure 15. The results show that the dialysis efficiency $\chi_{u}$ increased with the membrane sieving coefficient and ultrafiltration rate, but with decreasing the retentate phase flow rate due to the greater residence time to transport solutes through the membrane, as illustrated by Figure 15.

## 6. Conclusions

Two-dimensional mass-transfer mathematical formulations are developed using Happel’s free surface model to describe velocity profiles by performing the perturbation method with the use of appropriate stream functions. Thus, the solutions for conjugated mass-transfer equations were solved numerically by the Crank–Nicolson method. Furthermore, the experimental results and theoretical predictions show that the dialysis rate was significantly enhanced with the ultrafiltration operation, particularly for the operations under the higher ultrafiltration rate. Therefore, a maximum dialysis rate improvement up to about seven times (say 674.65% under $V_{a}=20\;\mathrm{mL}/\min)\;$was found in the operation of the hollow-fiber dialyzer with ultrafiltration rate $V_{w}=10\;\mathrm{mL}/\min$ and membrane sieving coefficient θ=1, compared with the system without ultrafiltration operation. The comparisons were made between the hollow-fiber dialyzer with/without ultrafiltration operations to draw the following conclusions:

1. The average outlet concentration of retentate phase increased with the retentate phase flow rate and ultrafiltration rate because the residence time decreased with dialysate phase flow rate, membrane sieving coefficient, and packing density.
2. The results show that the dialysis rate M increased with both retentate and dialysate phase flow rates, membrane sieving coefficient, ultrafiltration rate, and packing density with respect to the system without ultrafiltration operation.
3. The results indicate that the packing density had a significant influence on the mass transfer rate in the membrane dialysis operations, and thus, the dialysis efficiency $\chi_{u}$ increased with the packing density.
4. The dialysis efficiency $\chi_{u}$ increased with the dialysate phase flow rate, ultrafiltration rate, membrane sieving coefficient, and packing density but decreased with the retentate phase flow rate.
5. The dialysis rate improvements $I_{u}\;\left(\%\right)$ increased with the ultrafiltration rate and membrane sieving coefficient but decreased with retentate phase flow rate.

The results demonstrate the technical feasibility of the hollow-fiber membrane dialyzer with ultrafiltration operation removing solutes (contaminants) in membrane-based separation processes. In this paper, the perspective dialysis efficiency under ultrafiltration operation was examined by implementing various ultrafiltration rates in the hollow-fiber dialyzer. Therefore, further investigations on the alternative membrane sieving coefficients, membrane materials, and ultrafiltration rates are required to assess the economic consideration.

## Figures and Tables

**Table 1:** Comparisons of the convergence tolerance with the use of the Crank-Nicolson method (Φ=0.2, $\theta =1,\;V_{w}=5\;\mathrm{mL}/\min$, $V_{a}=40\;\mathrm{mL}/\min,$ $V_{b}=300\;\mathrm{mL}/\min)$.

Dimensionless Concentration	*P* = 100		*P* = 200	
	*N* = *J* = 200	*N* = *J* = 300	*N* = *J* = 200	*N* = *J* = 300
$\phi_{aw}$	0.89	0.89	0.89	0.89
$\bar{\phi_{a,o}}$	0.97	0.97	0.97	0.97
$\phi_{bw}$	0.36	0.36	0.36	0.36
$\bar{\phi_{b,o}}$	0.06	0.06	0.06	0.06

**Table 2:** Comparisons of the zero and first order perturbation methods for $u_{b\xi }$ (Φ=0.3, $\xi =0.5,\;V_{b}=100\;\mathrm{mL}/\min$).

η	$V_{w}=5\;mL/min$		$V_{w}=10\;mL/min$	
	Zero Order	First Order	Zero Order	First Order
0.42	0.58	0.58	0.59	0.59
0.70	1.11	1.11	1.14	1.14
1.0	1.61	1.61	1.65	1.65

**Table 3:** Calculated Reynolds number in retentate phase.

Φ	$Re_{a}$			$Re_{b}$		
	$V_{a}=20\;mL/min$	$V_{a}=30\;mL/min$	$V_{a}=40\;mL/min$	$V_{b}=100\;mL/min$	$V_{b}=200\;mL/min$	$V_{b}=300\;mL/min$
0.2	25.65	38.47	51.29	28.30	56.60	84.91
0.3	16.93	25.39	33.85	21.39	42.78	64.18
0.4	12.82	19.23	25.65	17.74	35.48	53.23

**Table 4:** Dialysis rate improvement $I_{u}\left(\%\right)$ of the hollow-fiber dialysis-and-ultrafiltration operation (Φ=0.2 and $V_{b}=300\;\mathrm{mL}/\min)$.

$V_{a}$ $\left(\mathrm{mL}/\min\right)$	θ=1		θ=0.6	
	$V_{w}=5\;\mathrm{mL}/\min$	$V_{w}=10\;\mathrm{mL}/\min$	$V_{w}=5\;\mathrm{mL}/\min$	$V_{w}=10\;\mathrm{mL}/\min$
20	296.35	674.65	259.04	554.41
30	223.56	535.70	186.71	405.89
40	183.07	457.15	147.01	327.63

**Table 5:** The percentage error deviation of the experimental results from the theoretical predictions.

$V_{a}$ (mL/min)	$V_{w}=0\;mL/min$			$V_{w}=5\;mL/min$		$V_{w}=10\;mL/min$			
	$\;\phi_{a,o}^{Exp}$	$\phi_{a,o}^{Theo}$	$Er\;\left(\%\right)$	$\phi_{a,o}^{Exp}$	$\phi_{a,o}^{Theo}$	$Er\;\left(\%\right)$	$\phi_{a,o}^{Exp}$	$\phi_{a,o}^{Theo}$	$Er\;\left(\%\right)$
20	0.899	0.938	4.32	0.937	0.972	3.79	0.987	0.980	0.66
25	0.906	0.953	5.18	0.944	0.982	4.01	0.991	0.984	0.24
30	0.909	0.949	4.42	0.954	0.980	2.68	0.992	0.987	0.50
35	0.910	0.953	4.76	0.958	0.982	2.53	1.016	0.989	2.65
40	0.903	0.956	5.89	0.973	0.984	1.01	1.022	0.990	3.11

## Data Availability

Data are contained within the article.

## Abbreviations

C	Solute concentration in the stream (mol m^−3^)
D	Solute diffusivity in both retentate and dialysate phases (m^2^ s^−1^)
$D_{m}$	Solute diffusivity in membrane (m^2^ s^−1^)
$I_{u}$	Dialysis rate improvement
J	Node numbers in η-direction of dialysate phase
L	Length of membrane dialyzer (m)
M	Dialysis rate (mol s^−1^)
N	Node numbers in η-direction of retentate phase
$N_{f}$	Number of fiber cells
P	Node numbers in ξ-direction
p	ξ-direction step sizes of the Crank–Nicolson method
$p_{a}$	Pressure in retentate phase, (N m^−2^)
$p_{b}$	Pressure in dialysate phase, (N m^−2^)
r	Transversal coordinate (m)
$r_{f}$	Imaginary free surface radius (m)
$r_{o}$	Outside radius of inner tube (m)
$r_{s}$	Inside radius of the shell tube (m)
$r_{t}$	Inside radius of inner tube (m)
Re	Reynolds number
$u_{a}$	Velocity distribution of the retentate phase (m s^−1^)
$u_{b}$	Velocity distribution of the dialysate phase (m s^−1^)
$\bar{u_{aI}}$	Averaged velocity of the retentate phase (m s^−1^)
$\bar{u_{bI}}$	Averaged velocity of the dialysate phase (m s^−1^)
$V_{aI}$	Volumetric flow rate of the retentate phase (m^3^ s^−1^)
$V_{bI}$	Volumetric flow rate of the dialysate phase (m^3^ s^−1^)
$V_{w}$	Ultrafiltration rate (m^3^ s^−1^)
$u_{w}$	Velocity distribution of the dialysate phase (m s^−1^)
$v_{w}$	Ultrafiltration flux distribution (m s^−1^)
$v_{w0}$	Average ultrafiltration flux on the inner surface of the membrane (m s^−1^)
$v_{w1}$	Average ultrafiltration flux on the outer surface of the membrane (m s^−1^)
z	Axial coordinate along the flow direction (m)
*Greek letters*	
$\Delta_{f}$	Ratio of inside radius of inner tube to imaginary free surface radius, $r_{t}/r_{f}$
$\Delta_{f1}$	Ratio of outside radius of inner tube to imaginary free surface radius, $r_{o}/r_{f}$
δ	Membrane thickness (m)
ε	Membrane porosity
η	Dimensionless transversal coordinate
θ	Membrane sieving coefficient
$\lambda_{a}$	Wall Reynolds number of the retentate phase, $\lambda_{a}=\Delta r_{s}v_{w0}/\upsilon$
$\lambda_{b}$	Wall Reynolds number of the dialysate phase, $\lambda_{b}=\Delta_{1}r_{s}v_{w1}/\upsilon$
μ	Wall Reynolds number of the dialysate phase (${\mathrm{kg}\;m}^{-1}{\;s}^{-1}$)
ξ	Dimensionless longitudinal coordinate
ρ	Density (${\mathrm{kg}\;m}^{-3}$)
υ	Kinetic viscosity ($m^{2}{\;s}^{-1}$)
ϕ	Dimensionless solute concentration
$\bar{\phi }$	Dimensionless average solute concentration
Φ	Packing density
$\chi_{u}$	Dialysis efficiency
φ	Stream function
*Subscripts*	
a	In the retentate phase
b	In the dialysate phase
I	At the inlet
o	At the outlet
u	Dialyzer module with ultrafiltration operation
w	On the membrane surface

## Appendix A

Substituting Equations (28) and (29) into Equations (5) and (6) in the main text, then, Equations (5) and (6) can be rewritten as

(A1) $$\;\left(\frac{4}{r_{f}^{2}}\right)\left[2\Delta^{2}\bar{u_{aI}}-4\frac{\Delta v_{w0}}{r_{f}}z\right]\left\{\Delta v_{w0}r_{f}\left(f_{a}f_{a}^{\prime \prime }-{\left(f_{a}^{\prime }\right)}^{2}\right)-\upsilon \left(\eta^{*}f_{a}^{\Delta }+f_{a}^{\prime \prime }\right)\right\}=-\frac{1}{\rho }\frac{\partial p_{a}}{\partial z}$$

and

(A2) $$\left(\frac{2\Delta v_{w0}}{r_{f}}\right)\;\left[\Delta v_{w0}r_{f}\left(\frac{2f_{a}f_{a}^{\prime }}{\eta^{*}}-\frac{f_{a}^{2}}{{\left(\eta^{*}\right)}^{2}}\right)-2\upsilon f_{a}^{\prime \prime }\right]=-\frac{1}{\rho }\frac{\partial p_{a}}{\partial \eta^{*}}$$

Combinations of the derivative of Equation (A1) with respect to r and the derivative of Equation (A2) with respect to *z* will obtain

(A3) $$\frac{\partial }{\partial \eta^{*}}\left\{\Delta v_{w0}r_{f}\left(f_{a}f_{a}^{\prime \prime }-{\left(f_{a}^{\prime }\right)}^{2}\right)-\upsilon \left(\eta^{*}f_{a}^{\Delta }+f_{a}^{\prime \prime }\right)\right\}=0$$

or

(A4) $$\eta^{*}f_{a}^{\Delta }+f_{a}^{\prime \prime }-\lambda_{a}\left[f_{a}f_{a}^{\prime \prime }-{\left(f_{a}^{\prime }\right)}^{2}\right]=k_{a}$$

In which $\lambda_{a}=\Delta r_{s}v_{w0}/\upsilon$ is the wall Reynolds number and $k_{a}$ is the integration constant. When the stream function of Equation (27) is introduced into the boundary conditions, Equations (9), (10), (14) and (25) can be rewritten as

(A5) $$f_{a}^{\prime \prime }\left(0\right)=0$$

(A6) $$f_{a}^{\prime }\left(\Delta^{2}\right)=0$$

(A7) $$f_{a}\left(0\right)=0$$

(A8) $$f_{a}\left(\Delta^{2}\right)=1/2$$

The perturbation method [30] was used to solve the expressions of both $k_{a}\;$and $f_{a}$ when $\left|\lambda_{a}\right|\leq 1$ as follows:

(A9) $$f_{a}=f_{a0}+\lambda_{a}f_{a1}+\lambda_{a}^{2}f_{a2}+\ldots +\lambda_{a}^{n}f_{an}+\ldots$$

(A10) $$k_{a}=S_{a0}+\lambda_{a}S_{a1}+\lambda_{a}^{2}S_{a2}+\ldots +\lambda_{a}^{n}S_{an}+\ldots$$

Applying perturbation method to Equations (A9) and (A10) with the use of boundary conditions yields $f_{a}\;$and the derivative$\;f\prime_{a}$ once all the constants ($f_{ai}$ and $k_{ai}$, i=0, 1, 2,…) were obtained as follows:

(A11) $$\begin{array}{cc} f_{a}= & \frac{1}{\Delta^{4}}\left(\Delta^{2}\eta^{*}-\frac{1}{2}{\left(\eta^{*}\right)}^{2}\right)+\lambda_{a}\left[\frac{\eta^{*}}{18\Delta^{2}}-\frac{{\left(\eta^{*}\right)}^{2}}{8\Delta^{4}}+\frac{{\left(\eta^{*}\right)}^{3}}{12\Delta^{6}}-\frac{{\left(\eta^{*}\right)}^{4}}{72\Delta^{8}}\right]\\ \; & \;+\lambda_{a}^{2}\left[\frac{83\eta^{*}}{5400\Delta^{2}}-\frac{19{\left(\eta^{*}\right)}^{2}}{540\Delta^{4}}+\frac{11{\left(\eta^{*}\right)}^{3}}{432\Delta^{6}}-\frac{{\left(\eta^{*}\right)}^{4}}{144\Delta^{8}}+\frac{{\left(\eta^{*}\right)}^{5}}{720\Delta^{10}}-\frac{{\left(\eta^{*}\right)}^{6}}{10800\Delta^{12}}\right] \end{array}$$

(A12) $$\begin{array}{cc} f_{a}^{\prime } & =\frac{\left(\Delta^{2}-\eta^{*}\right)}{\Delta^{4}}+\lambda_{a}\left[\frac{1}{18\Delta^{2}}-\frac{\eta^{*}}{4\Delta^{4}}+\frac{{\left(\eta^{*}\right)}^{2}}{4\Delta^{6}}-\frac{{\left(\eta^{*}\right)}^{3}}{18\Delta^{8}}\right]\\ \; & +\lambda_{a}^{2}\left[\frac{83}{5400\Delta^{2}}-\frac{19\left(\eta^{*}\right)}{270\Delta^{4}}+\frac{11{\left(\eta^{*}\right)}^{2}}{144\Delta^{6}}-\frac{{\left(\eta^{*}\right)}^{3}}{36\Delta^{8}}+\frac{{\left(\eta^{*}\right)}^{4}}{144\Delta^{10}}-\frac{{\left(\eta^{*}\right)}^{5}}{1800\Delta^{12}}\right] \end{array}$$

The dimensionless forms of velocity distributions, $u_{a\xi }$ and $u_{a\eta }$, of Equations (7) and (8) were obtained after substituting Equations (A11) and (A12) into Equations (28) and (29) accordingly.
